# Investigation of the Effect of Periodontitis on Trabecular Bone Structure by Fractal Analysis

**DOI:** 10.7759/cureus.77833

**Published:** 2025-01-22

**Authors:** Emrullah B Dosdogru, Melis Ziyaettin, Ahmet F Ertürk

**Affiliations:** 1 Periodontology, Biruni University, İstanbul, TUR; 2 Oral and Maxillofacial Radiology, Biruni University, İstanbul, TUR

**Keywords:** digital radiography, fractal analysis, fractal dimension, panoramic radiography, periodontitis

## Abstract

Objective: This study aimed to evaluate the trabecular microarchitecture in the interdental region of panoramic radiographs from periodontally healthy individuals and those with stage III/IV periodontitis using fractal analysis (FA), while also assessing the relationship between clinical periodontal parameters and FA outcomes.

Study design: Clinical and radiographic records of 20 periodontally healthy individuals and 19 individuals with stage III/IV periodontitis were included. Clinical measurements including the plaque score, bleeding on probing, probing pocket depth, and clinical attachment level were recorded. A region of interest was selected on panoramic radiographs from the mesial aspect of the mandibular first molar and the distal aspect of the second premolar. The fractal dimension (FD) values for this region were calculated using the box-counting method. The statistical significance of the difference was evaluated at P<0.05.

Results: There was no significant statistical difference in sex between the groups (p > 0.05). Conversely, individuals in the periodontitis group had a significantly higher mean age compared to those in the healthy group (p < 0.01). Clinical measurements were significantly higher in the periodontitis group (p < 0.01). However, no significant differences in FD values were observed between the groups (p > 0.05). In the healthy group, moderate negative correlations were found between FD and clinical parameters (with all p < 0.05), while no such correlations were identified in the periodontitis group (with all p > 0.05).

Conclusion: FA demonstrates potential as a complementary diagnostic tool when used alongside clinical periodontal measurements. However, its effectiveness as a standalone method for reliably distinguishing between healthy individuals and those with periodontitis remains limited. Advanced radiographic analysis can further enhance the identification of anatomical details, thereby improving the accuracy of periodontal evaluations and diagnostics.

## Introduction

Periodontal diseases are chronic infections and a leading cause of tooth loss, with their development influenced by multiple contributing factors [1]. While the primary causative agent is the pathogenic microbial biofilm, the progression and clinical manifestation of the disease are predominantly driven by the host's immune response and individual characteristics [2].

The severity of periodontitis is assessed using direct evidence, such as radiographic bone loss or attachment loss over a five-year period, and indirect evidence, including case phenotype and the percentage of bone loss [3]. In addition, it incorporates secondary criteria, such as risk factors like smoking and diabetes, inflammatory burden, and biomarker indicators of bone loss [3,4]. Alveolar bone changes can reflect the onset and progression of periodontitis. Therefore, evaluating alterations in alveolar bone structure is one of the essential methods for preventing periodontal diseases, planning treatment, and determining prognosis. Radiography is a vital tool used at various stages of periodontal assessment. Different imaging techniques, such as panoramic radiography, intraoral radiography, and cone-beam computed tomography, can be utilized to monitor the condition of periodontal tissues [5-7]. However, bone changes typically become visible on radiographs only after more than 30% of the bone mineral content has been resorbed [8]. Additional analysis of radiographs could enhance their diagnostic value by improving the detection of details.

Fractal analysis (FA) can serve as an alternative method for quantitatively assessing trabecular changes in alveolar bone structure. This technique identifies intricate structural patterns and expresses them numerically as the fractal dimension (FD) [9]. When calculated using radiographs, FD provides a measure of the complexity of the alveolar bone surrounding the teeth [10]. FA is primarily applied to digital radiographs, generating an FD value for the image. This value represents how much the object occupies space and describes its self-similarity. Among the various methods to calculate FD, the box-counting technique introduced by White and Rudolph is the most widely used and is particularly effective for analyzing binary images [11,12].

According to the available evidence, a limited number of studies have investigated trabecular changes in the alveolar bone of periodontitis patients using FA, and conflicting results have been reported due to the lack of standardization [13,14]. This study aimed to evaluate the effect of periodontal disease on trabecular bone density using FA, examine its ability to detect trabecular changes in periodontitis, and explore its relationship with clinical periodontal measurements. The null hypothesis (H₀) of the present study states that “there is no significant difference in FD measurements between the healthy and periodontitis groups”.

## Materials and methods

This study was conducted in accordance with the principles of the Declaration of Helsinki and approved by the Ethics Committee of Biruni University (2024-BİAEK/05-50). Informed consent was obtained from all individuals. The study included 20 periodontally healthy individuals and 19 patients with stage III/IV periodontitis. Periodontal status was confirmed through dental history, clinical symptoms, clinical records, and radiographic findings. In the healthy group, individuals meeting specific criteria to ensure periodontal health were included in the study [15]. Suitable individuals had no history of periodontal disease and exhibited clinically and radiographically healthy periodontal tissues. Additional inclusion criteria, consistent with established definitions of periodontal health, required a bleeding on probing (BOP) score of less than 10% and a probing pocket depth (PPD) ≤3 mm.

Patients diagnosed with stage III or IV periodontitis [3] were included based on specific clinical and radiographic criteria. These criteria included interdental clinical attachment loss of ≥5 mm in at least two non-adjacent teeth and a history of tooth loss attributed to periodontal disease. Radiographic findings further confirmed the diagnosis, showing bone loss extending to the middle or apical third of the root. The choice of the relevant site was based on the study that showed that bone loss measurements obtained from mandibular posterior teeth are optimal for periodontal disease index [14].

Individuals were excluded from the study if they met any of the following criteria. Exclusion criteria related to systemic conditions included a history of systemic diseases known to affect periodontal health, such as diabetes or osteoporosis, and the use of medications that could influence periodontal status, such as bisphosphonates, immunosuppressants, or corticosteroids. Radiographic exclusions included cases with low image quality due to poor resolution or technical artifacts. Dental exclusions encompassed the presence of jaw pathologies such as tumors or developmental anomalies, teeth with deep cervical caries, previously root-treated teeth, and the presence of periapical lesions. Individuals with missing left mandibular the first molar and/or second premolar were excluded from the study. 

All clinical measurements were conducted by two calibrated assessors (E.B.D. and M.Z.). Inter-examiner reproducibility was assessed by examining 10 individuals (five periodontally healthy and five with periodontitis) who were not included in the study. PPD measurements were repeated independently by both assessors at six sites per tooth after a 48-hour interval. The intraclass correlation coefficient (ICC) was 0.998 (0.994-1), indicating excellent intra-examiner reproducibility. Plaque score (PS), PPD, BOP, and clinical attachment level (CAL) were measured at six sites per tooth (mesio-buccal, mid-buccal, disto-buccal, mesio-lingual, mid-lingual, and disto-lingual) using a periodontal probe (Hu-Friedy, Chicago, IL, USA). FD analyses of the radiographs were performed by a second examiner (A.F.E.), who did not perform the clinical measurements.

Radiographic analysis

**Figure 1:**
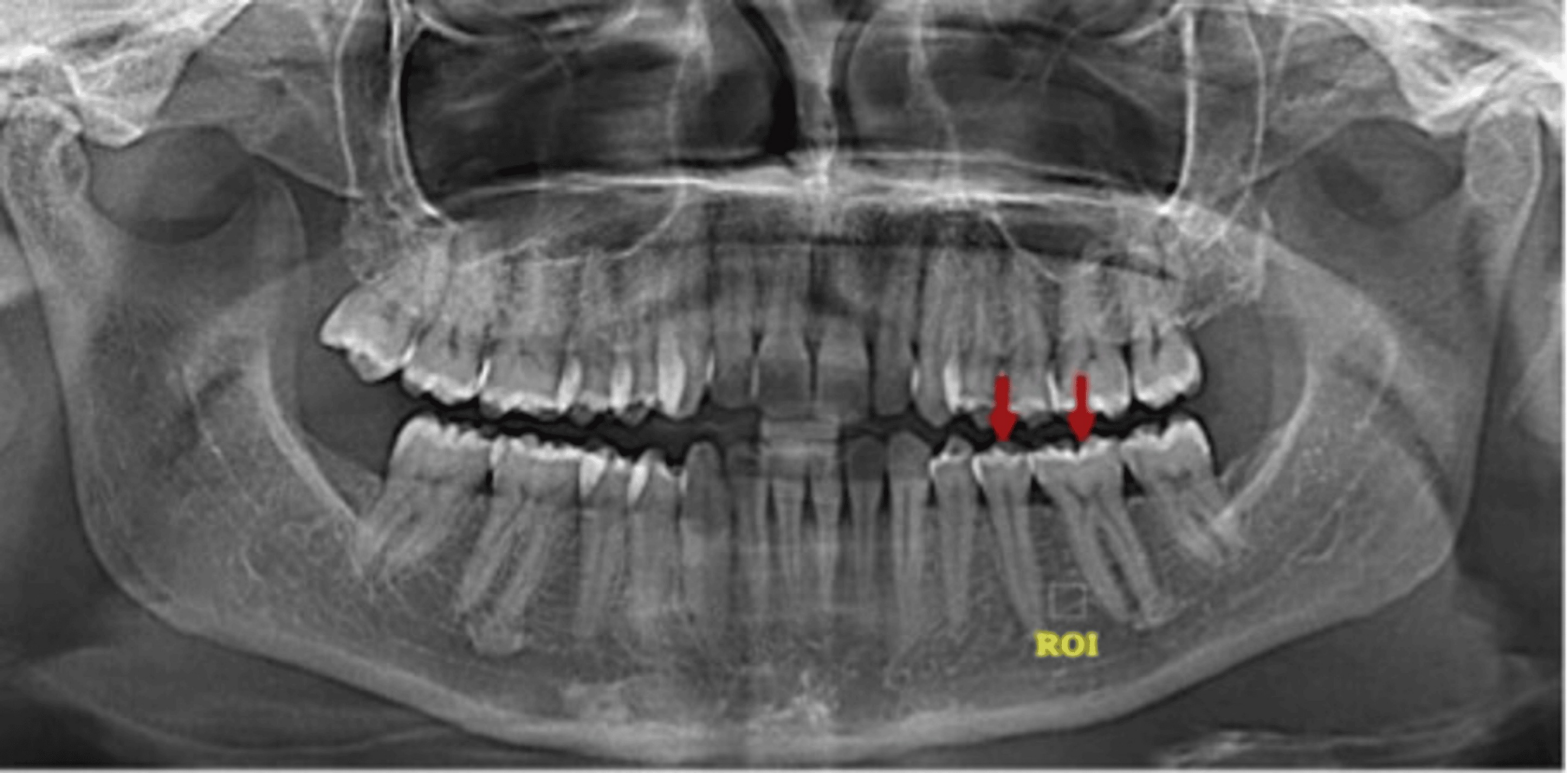
The mesial and distal regions were represented by uniform rectangular regions of interest (ROIs) placed along the margins of healthy trabecular bone.

All digital panoramic radiographs were obtained using the same device (Sirona, Germany; 68 kVp, 8 mA, 14-second exposure time). Bone trabeculation was analyzed using “Image J” software (National Institutes of Health, USA). Radiographic images were processed via the software’s image-processing functions, and numerical values of the trabecular structure were calculated using FA. Standard regions of interest (ROIs) of 30 x 30 pixels were selected between the roots of the left mandibular first molar and second premolar (Figure 1).

**Figure 2:**
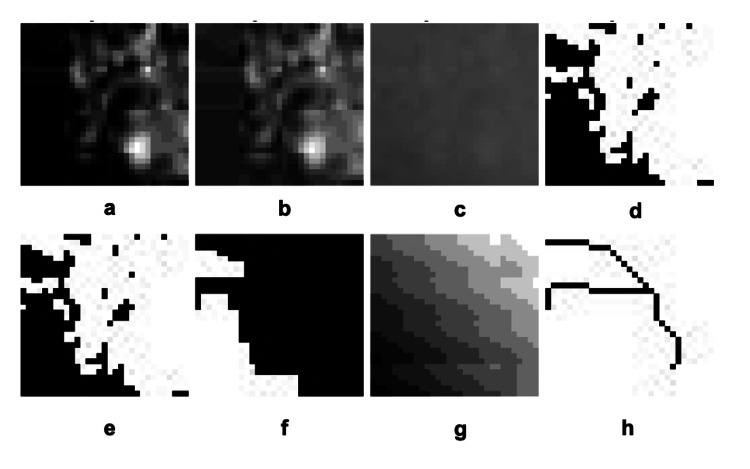
Steps used in image processing. a. Blur: The image was blurred. b. Subtract: The contrast was reduced, and details were extracted. c. Adding 128 shades of gray: Shades of gray were added. d. Threshold: The image was binarized using thresholding. e. Erode: Black areas were expanded (erosion process). f. Dilate: White areas were expanded (dilation process). g. Color Inversion: The colors were inverted. h. Skeleton image: The image was reduced to its skeleton structure.

FD values were calculated using the box-counting method (Figure 2) [11]. During image optimization, a Gaussian filter was applied to a copy of the original image to create a blurred version, which was then subtracted from the original image. A gray value of 128 was added to each pixel, and the resulting image was converted to binary for black-and-white visualization. Erosion, dilation, and skeletonization functions were subsequently applied. Finally, the FD of the selected ROI was determined.

Statistical analysis

Data obtained in the study were analyzed using IBM SPSS Statistics for Windows, Version 27.0 (released 2020, IBM Corp., Armonk, NY). Quantitative variables were presented using descriptive statistical methods, such as mean, standard deviation, median, minimum, and maximum values, while qualitative variables were expressed as frequencies and percentages. The normality of data distribution was assessed using the Shapiro-Wilk test and box-plot graphics. For normally distributed variables, comparisons between two groups were performed using the Student’s t-test, while the Mann-Whitney U test was used for comparisons of non-normally distributed variables. The Chi-square test was used for comparisons of categorical data. Spearman’s correlation analysis was conducted to evaluate relationships between variables. Results were interpreted at a 95% confidence interval, with statistical significance set at p < 0.05.

## Results

A total of 39 individuals participated in the study, comprising 56.4% females (n = 22) and 43.6% males (n = 17), including 20 healthy individuals and 19 with periodontitis. The participants' ages ranged from 20 to 53 years, with a mean age of 35.59 ± 9.92 years. There was no statistically significant difference in sex distribution between the groups (p > 0.05). The age of individuals in the periodontitis group was significantly higher compared to the healthy group (p = 0.001; p < 0.01) (Table 1). 

**Table 1:** Demographic characteristics of the groups. The data presented in this table are described using specific statistical parameters for enhanced clarity and interpretability. "SD" denotes the standard deviation, which reflects the variability of the data around the mean. "Min" and "Max" represent the minimum and maximum values observed in the dataset, respectively, providing an overview of the range. "N" refers to the sample size or the number of observations included in the analysis. Statistically significant results are highlighted in bold to draw attention to meaningful differences or associations. The superscripts "a" and "b" indicate the statistical tests employed, with "a" denoting the Pearson chi-square test and "b" denoting the Student’s t-test. Results deemed statistically significant are marked with three asterisks (***), indicating a significance threshold of p < 0.001.

		Group				p
		Healthy (N = 20)		Periodontitis (N = 19)		
Sex	Female	N: 12	%60.0	N: 10	%52.6	^a^0.643
	Male	N: 8	%40.0	N: 9	%47.4	
Age	Mean ± SD	29.70 ± 8.71		41.79 ± 6.98		^b^0.001**
	Median (Min-Max)	27.5 (20-53)		43 (28-53)		

The FD values did not exhibit any significant differences between patients with periodontitis and healthy individuals. However, all clinical periodontal measurements were found to be significantly higher in the periodontitis group compared to the healthy group (p = 0.001; p < 0.01) (Table 2). There was no statistically significant correlation observed between FD values and age in either the periodontitis or healthy groups (r = -0.133, p = 0.587; r = 0.061, p = 0.799, respectively). In terms of FD, there were no statistically significant differences between males and females in either group (p > 0.05). 

**Table 2:** Comparison of clinical periodontal measurements between groups. The data in this table are represented using various statistical parameters to enhance clarity and provide detailed insights into the results. "SD" stands for standard deviation, reflecting the degree of variability within the data. "Min" and "Max" denote the minimum and maximum values observed in the dataset, respectively, providing an understanding of the data range. "N" indicates the number of observations or sample size, while "FD" represents the fractal dimension, a parameter describing complex structures. "PS" refers to the plaque score, "PPD" represents the probing pocket depth, "CAL" denotes the clinical attachment level, and "BOP" stands for bleeding on probing. Statistically significant results are highlighted in bold to emphasize key findings. The statistical methods applied are identified by superscripts, where "b" represents the Student’s t-test and "c" indicates the Mann-Whitney U test. Statistical significance is further denoted by double asterisks (**), indicating a significance level of p < 0.01, underscoring the reliability of the observed differences or relationships within the data.

		Group		p
		Healthy (N = 20)	Periodontitis (N = 19)	
FD	Mean ± SD	1257.40 ± 70.64	1227.79 ± 69.79	^b^0.196
	Median (Min-Max)	1261 (1104-1377)	1235 (1088-1375)	
PS (%)	Mean ± SD	9.48 ± 6.02	68.56 ± 20.37	^c^0.001**
	Median (Min-Max)	8,9 (1.1-24.4)	67.4 (32-100)	
PPD (mm)	Mean ± SD	1.95 ± 0.39	3.65 ± 0.79	^c^0.001**
	Median (Min-Max)	2 (1.4-3.2)	3,7 (2.2-5.7)	
CAL (mm)	Mean ± SD	1.96 ± 0.39	3.99 ± 0.94	^c^0.001**
	Median (Min-Max)	2 (1.4-3.2)	3.9 (2.7-5.9)	
BOP (%)	Mean ± SD	5.75 ± 2.07	59.55 ± 21.52	^c^0.001**
	Median (Min-Max)	6 (2.9-9.5)	60.2 (25.3-100)	

The correlations between FD and clinical periodontal measurements for each group are presented in Table 3. A moderate, negative, and statistically significant correlation was observed between FD and PS, PPD, CAL, and BOP in the healthy group (r = 0.576, p = 0.008; r = -0.540, p = 0.014; r = -0.515, p = 0.020; and r =- 0.490, p = 0.028, respectively). By contrast, no statistically significant correlations were found between FD and PS, BOP, PPD, or CAL in the periodontitis group (p > 0.05).

**Table 3:** Correlation between fractal dimension and clinical periodontal measurements between groups. The data presented in this table are described using various parameters to enhance clarity and provide a detailed understanding of the statistical findings. "FD" represents the fractal dimension, used to characterize complex structures. "BOP" denotes bleeding on probing, "PS" refers to plaque score, "PD" represents probing depth, and "CAL" indicates clinical attachment level. Statistically significant results are highlighted in bold font to emphasize key findings. The statistical method employed is indicated by "r," which represents the Spearman correlation test. Double asterisks (**) denote a significance level of p < 0.01, indicating that the observed relationships are statistically reliable.

Group	Clinical periodontal measurements	r/p	FD
Healthy	PS	r	-0.576
		p	0.008**
	PPD	r	-0.540
		p	0.014*
	CAL	r	-0.515
		p	0.020*
	BOP	r	-0.490
		p	0.028*
Periodontitis	PS	r	0.032
		p	0.896
	PPD	r	0.066
		p	0.789
	CAL	r	-0.014
		p	0.954
	BOP	r	-0.090
		p	0.713

## Discussion

Periodontitis is a condition that can present in either acute or chronic forms and is influenced by various etiological factors [1]. As periodontal infections progress, they often result in alveolar bone destruction and tooth loss, which can have significant negative effects on patients. This disease affects a large portion of the global population. It has been estimated that 11.2% of individuals worldwide are affected by this condition [16]. Identifying bone loss at an early stage can help prevent further progression and related complications. Improving the analysis of medical images plays a vital role in various diagnostic applications [8]. Detecting differences in bone structures on FA radiographs using mathematical morphology provides a potential approach to enhance the diagnostic accuracy of radiographs [17]. Studies have utilized varying exposure times (mAs), peak kilovoltage (kVp), and projection angles for FD analysis, indicating the absence of a standardized protocol [18-20].

According to Kato's 2020 meta-analysis, the box-counting method was identified as the most widely used and current approach in FA [21]. This method adheres to the procedural steps introduced by White and Rudolph and is supported by the Image J software, developed specifically for image analysis. In alignment with previous studies, our research utilized the box-counting method in the ImageJ software (Rasband, W.S., ImageJ, U. S. National Institutes of Health, Bethesda, Maryland, USA, https://imagej.net/ij/, 1997-2018) to perform FA [6,22].

This study aimed to evaluate FD in periodontitis and healthy individuals using panoramic radiographs, assess the potential contribution of the FA method to radiographic diagnosis, and examine the possible relationship of FD with clinical periodontal measurements. The results did not disprove the null hypothesis, suggesting that there is no statistically significant difference in FD values between individuals with periodontitis and healthy individuals.

FA can provide additional information on factors such as bone loss and contribute to risk assessment alongside clinical periodontal measurements [4]. Evaluating FA in maintenance-oriented periodontal therapies, such as frequent monitoring of patients with low FA values and improving oral hygiene, may be beneficial. Coşgunarslan et al. [22] analyzed FD values derived from CBCT images in healthy individuals and periodontitis patients, reporting no statistically significant differences between the two groups, similar to the findings of our study. However, the detection of significant correlations between FA and certain clinical periodontal measurements suggests that FA could make an important contribution to the diagnostic process when compared to traditional clinical assessments. The lack of significant differences in FA values between periodontitis and healthy groups may be attributed to the insufficient sensitivity of FA in detecting minor alveolar bone changes during the progression of periodontitis.

In the literature, studies have evaluated the trabecular structure of bone using FA with panoramic [23,24] and periapical radiographs [19,25]. In their study measuring FD values using periapical radiographs, Sener et al. [26] found a significant difference between healthy individuals and patients with moderate periodontitis. Bollen et al. [17], in their comparative analysis of periapical and panoramic radiographs, reported that FD values obtained from periapical radiographs of the same patients were significantly higher. This finding suggests that variations in imaging methods, such as differences in detail and resolution, may influence the outcomes, particularly in diseases like periodontitis that cause localized bone loss. Moreover, selecting an identical ROI from the same region in every patient is not feasible, which may result in measurement discrepancies [14]. This variability in measurement methodologies is likely a contributing factor to the inconsistencies observed in the results reported in the literature. Consistent with the literature, our study also found no significant relationship between sex and FD [6,9,27]. Variations in mandibular size, shape, medical history, and bone metabolism dynamics may overshadow gender-specific effects. A larger sample size is required to better understand the impact of sex on trabecular bone architecture. In our study, although the periodontitis group was older than the healthy group, no correlation was found between FD and age or sex in either group. Further studies with larger sample sizes are needed to determine whether these factors play a role in periodontal health. In the absence of studies in the literature employing a methodology directly comparable to ours, it is challenging to draw direct comparisons regarding the relationship between our clinical periodontal measurements and FD values.

In this study, panoramic radiographs were selected for FA analysis based on the As Low As Reasonably Achievable (ALARA) principle, prioritizing patient safety while considering lower radiation dose, cost-effectiveness, and accessibility. Alternatively, 2D radiographs provide sufficient diagnostic quality with significantly lower doses for specific indications. Compared to computed tomography images, 2D plain radiographs offer higher spatial resolution and can reveal morphological changes in bone trabeculation compared to controls. In this context, FA on 2D imaging stands out as a non-invasive method in dental radiology for quantifying trabecular bone [21,28].

Various factors, including patient selection, imaging quality, sample size, sex, age distribution across groups, ROI location and size variability, individual differences in ROI selection, and anatomical diversity among patients, are likely to influence study outcomes. To mitigate the effects of these factors, it is essential to address methodological limitations and conduct further studies with larger, more standardized sample sizes.

## Conclusions

In this study, FD values and clinical periodontal measurements of individuals with periodontitis, and healthy controls were compared using panoramic radiographs. The analysis revealed no statistically significant difference in trabecular bone FD values between the periodontitis and healthy groups. In addition, FD values showed no significant correlation with gender or age in either group.

Notably, in the healthy group, moderate negative correlations were observed between FD and clinical parameters, including PS, PPD, CAL, and BOP. By contrast, no significant correlations were found between FD and clinical measurements in the periodontitis group. Based on the results of this study, FD does not appear to have sufficient diagnostic value for distinguishing between healthy and periodontitis cases. Further research with larger sample sizes and standardized methodologies is required to clarify the potential role of FD in periodontal assessments.
